# Third generation cephalosporins and piperacillin/tazobactam have distinct impacts on the microbiota of critically ill patients

**DOI:** 10.1038/s41598-021-85946-4

**Published:** 2021-03-31

**Authors:** Hasinika K. A. H. Gamage, Carola Venturini, Sasha G. Tetu, Masrura Kabir, Vineet Nayyar, Andrew N. Ginn, Belinda Roychoudhry, Lee Thomas, Mitchell Brown, Andrew Holmes, Sally R. Partridge, Ian Seppelt, Ian T. Paulsen, Jonathan R. Iredell

**Affiliations:** 1 Department of Molecular Sciences, Macquarie University, Sydney, NSW Australia; 2 Centre for Infectious Diseases and Microbiology, The Westmead Institute for Medical Research, The University of Sydney and Westmead Hospital, Sydney, NSW Australia; 3 Intensive Care Unit, Westmead Hospital and The University of Sydney, Sydney, NSW Australia; 4 Centre for Infectious Diseases and Microbiology Laboratory Services, NSW Health Pathology, Sydney, NSW Australia; 5 Charles Perkins Centre, The University of Sydney and Westmead Hospital, Sydney, NSW Australia; 6 Department of Intensive Care Medicine Unit, Nepean Hospital, Sydney University Medical School (Nepean), The University of Sydney, Sydney, NSW Australia; 7 Department of Clinical Medicine, Macquarie University, Sydney, NSW Australia; 8 Present Address: Westmead Breast Cancer Institute, Westmead Hospital, Sydney, NSW Australia

**Keywords:** Microbiology, Molecular biology, Medical research

## Abstract

Effective implementation of antibiotic stewardship, especially in critical care, is limited by a lack of direct comparative investigations on how different antibiotics impact the microbiota and antibiotic resistance rates. We investigated the impact of two commonly used antibiotics, third-generation cephalosporins (3GC) and piperacillin/tazobactam (TZP) on the endotracheal, perineal and faecal microbiota of intensive care patients in Australia. Patients exposed to either 3GC, TZP, or no β-lactams (control group) were sampled over time and 16S rRNA amplicon sequencing was performed to examine microbiota diversity and composition. While neither treatment significantly affected diversity, numerous changes to microbiota composition were associated with each treatment. The shifts in microbiota composition associated with 3GC exposure differed from those observed with TZP, consistent with previous reports in animal models. This included a significant increase in *Enterobacteriaceae* and *Enterococcaceae* abundance in endotracheal and perineal microbiota for those administered 3GC compared to the control group. Culture-based analyses did not identify any significant changes in the prevalence of specific pathogenic or antibiotic-resistant bacteria. Exposure to clinical antibiotics has previously been linked to reduced microbiota diversity and increased antimicrobial resistance, but our results indicate that these effects may not be immediately apparent after short-term real-world exposures.

## Introduction

The human gut microbiota plays an integral role in host metabolic functions and immunity. Intensive care has been associated with substantial changes in the intestinal microbiota of patients, including reduced microbial diversity, altered composition with a propensity for expansion of pathobionts such as Proteobacteria, and increased levels of antibiotic resistance^1–3^. However, the magnitude of these changes is not uniform among individuals due to large interpersonal variation in baseline microbiota composition^4,5^. Antibiotics are heavily used in intensive care units (ICUs) and timely antibiotic intervention, immediately upon presentation, saves lives^6^. Antimicrobial exposure, however, can have long-lasting detrimental impacts on gut microbiota health, including reduced resistance against subsequent infections^7–9^, with recovery from dysbiosis dependent on the type of antibiotic treatment received^5,10–12^. There is therefore urgent need to define ways to preserve microbiota integrity in ICU patients, including the use of probiotics and selective digestive tract decontamination to reduce microbiota dysbiosis^13–15^.

Antibiotic stewardship programs aim to reduce unnecessary and inappropriate antimicrobial use through optimal therapeutic choices^16,17^. Antibiotic regimens in critical care typically include broad-spectrum drugs targeting the main nosocomial pathogens^18,19^, and associated colonisation by antibiotic-resistant bacteria and perturbations of gut microbiota composition and functions are largely predictable^20,21^. However, different antibiotics vary in the spectrum of activity, route of administration, pharmacokinetics (distribution and elimination in the host) and pharmacodynamics (interactions between drug and pathogen)^21–23^. Some antibiotics may be more harmful to the microbiota than others^20,22^.

Third-generation cephalosporins (e.g. ceftriaxone, cefotaxime; 3GC) and penicillin/β-lactamase inhibitor combinations (piperacillin/tazobactam; TZP) are broad-spectrum antimicrobials commonly used in critical care^24^. They have very similar pharmacokinetic and pharmacodynamic characteristics and spectra of activity against medically important pathogens but data from animal models^12,23,25^ and accumulated clinical experience suggest different ecological outcomes^3,26,27^. Both antibiotic classes are associated with an increased presence of antibiotic-resistant *Enterobacteriaceae,* staphylococci*, Clostridioides difficile* and *Pseudomonas aeruginosa* in the microbiota^28–30^ but 3GC has been shown to result in poorer microbiota recovery and longer-lasting changes^12,31^ and antibiotic guidelines often recommend restricted use due to concerns linked to ‘collateral damage’ to the commensal microbiota^32^. Despite this, there is a lack of direct comparative data. We, therefore, examined the differential impact of 3GC and TZP on the endotracheal, perineal and faecal microbiota of ICU patients in two hospitals in Sydney, Australia.

## Results and discussion

The impact of administrating 3GC or TZP on the endotracheal, perineal and faecal microbiota composition was examined using 16S rRNA amplicon sequencing. A total of 222 samples were collected over time (from 24 h to 11 days after ICU admission) from patients receiving either 3GC, TZP or no β-lactams (control group), details on the number of samples collected per treatment group and patient are provided in Supplementary Table S1. All patients in the 3GC and TZP groups received the respective antibiotics within the first 48 h of ICU admission. None of the patients enrolled in the study had exposure to antibiotics in the two weeks before ICU admission.

### Each body site had a unique initial microbial composition

Samples collected within 48 h of antibiotic administration (for 3GC and TZP groups) or ICU admission (for control group) were considered representative of the initial microbiota of each patient. This classification was based on previous publications where no significant change in the gut microbiota or antibiotic resistance was observed within the first 48 h of antibiotic exposure^3,12,33^. The exact microbial composition in each body site varied significantly between individuals (Supplementary Fig. S1). The initial microbiota of patients receiving 3GC or TZP had differences in the relative abundance of some bacterial families compared to those in the control group (Supplementary Table S2), for example, the abundance of *Enterobacteriaceae* was significantly higher in the initial endotracheal microbiota of the 3GC group compared to the control group. This could be due to different underlying infections of patients in the 3GC and TZP groups compared to those receiving neither antibiotics (control group) or may be indicative of microbiota changes in the early stages of antibiotic therapy. Limitations inherent to real-world ICU studies, including a lack of samples collected before illness or antibiotic administration and the variable availability of some samples (especially faecal samples) add to difficulties in interpretation.

The initial composition of the perineal microbiota showed a simpler community composition with > 90% of taxa belonging to a single family, *Staphylococcaceae*, in 13 out of 24 samples collected within the first 48 h (Supplementary Fig. S1), similar to previous reports showing a simpler microbial community in the perineal microbiota^34^. In contrast, the other two body sites had microbiota composition distributed across three or more bacterial families. The endotracheal microbiota was dominated by the *Pasteurellaceae*, *Streptococcaceae* and *Enterobacteriaceae*^35–37^, each family contributing to > 10% of the total microbial abundance in at least 11 out of 39 initial endotracheal microbiota samples collected. Some members of these families represent potential pathogens in the lower respiratory tract^38,39^. The faecal microbiota samples had a higher abundance of obligate anaerobic gut commensal bacteria such as *Bacteroidaceae* and *Lachnospiraceae*^40^, each of these two families contributed to > 10% of the microbial abundance in at least 9 out of 24 faecal samples collected within the first 48 h of ICU admission (control group) or antibiotic exposure (3GC and TZP).

### Overall microbiota community structure and diversity were not significantly impacted by either antibiotic treatment

**Figure 1:**
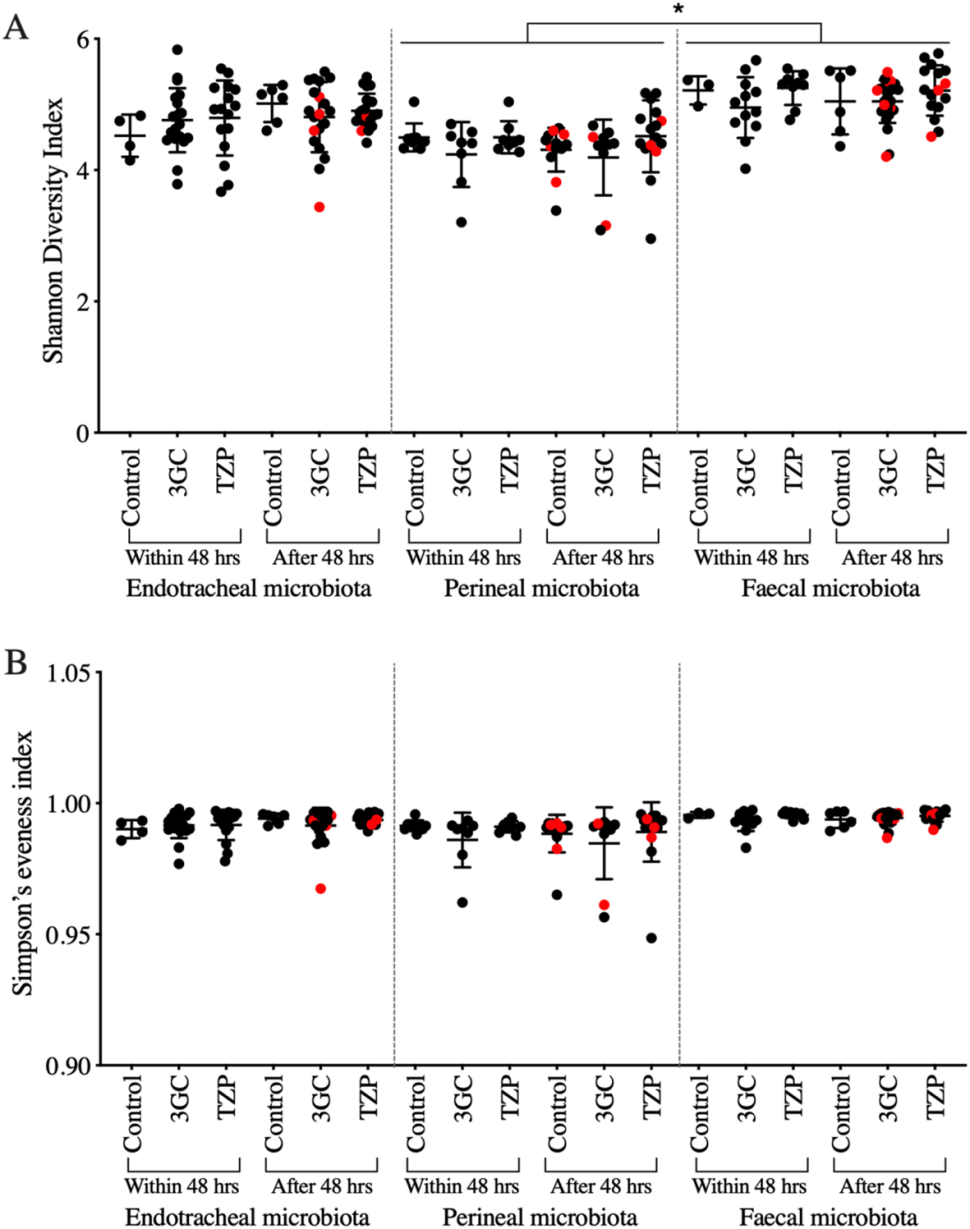
Alpha diversity of microbial communities of the 3GC, TZP and control groups presented as (**A**) Shannon diversity and (**B**) Simpson’s evenness indices, (3GC-third-generation cephalosporins, TZP-piperacillin/tazobactam, control-no β-lactams). Both alpha diversity indices were determined using microbial abundance at the OTU level. Data are shown as mean ± standard deviation. Dots represent individual samples for endotracheal, faecal and perineal microbiota of each patient. Samples collected within and after 48 h of antibiotic administration (3GC and TZP groups) or ICU admission (control group) are shown. Diversity indices of microbiota samples collected between day 6 and 11 are shown in red. Significance was determined by Tukey’s multiple comparison tests with Geisser-Greenhouse correction (* *P* < 0.05) using GraphPad Prism (version 9, GraphPad Software, USA, www.graphpad.com). Detailed information on sample size per treatment group are provided in Supplementary Table S1.

The impact of the two antibiotic regimens on the overall microbiota community structure was examined using multi-dimensional scaling (MDS) plots based on Bray–Curtis similarity metrics of the operational taxonomic unit (OTU) abundance. These revealed no statistically significant difference in the overall community structure of the initial microbiota (< 48 h) between the three groups and neither antibiotic treatment resulted in a significant shift in the overall microbiota community in any of the three body sites after 48 h compared that in the control group (Supplementary Fig. S2). Integrated metrics of microbiota alpha diversity, Shannon diversity and Simpson’s evenness indices, showed that perineal microbiota diversity was lower than the faecal microbiota diversity (Fig. 1). However, microbiota alpha diversity in neither of these sites was significantly different to that of the endotracheal microbiota. The two antibiotic treatments were not associated with a significant change in the microbiota diversity over the course of this study; there was no significant difference in the diversity or evenness indices in any of the body sites when samples collected > 48 h after antibiotic administration were compared against those collected < 48 h of antibiotic administration or the control group. Samples collected from day 6 to day 11 showed no significant change in alpha diversity compared to the control group and those collected within the first 48 h of antibiotic therapies, indicating no significant impact of longer-term administration of 3GC or TZP on microbiota diversity of the three body sites. The alpha diversity indices in all samples were comparable to that of other microbiota studies conducted on healthy adults^5,41^, indicating a generally diverse overall microbiota community. Compared to the control group, there was no significant difference in microbiota alpha diversity in those receiving either 3GC or TZP. Antimicrobial therapy, especially with broad-spectrum antibiotics, has previously been associated with a lower alpha diversity in the gut microbiota^1,5,42^. In this study, however, neither antibiotic regimen significantly altered overall microbial alpha-diversity in any of the three body sites, suggesting that the use of powerful broad-spectrum β-lactams does not immediately result in a strong decline in microbiota diversity relative to other ICU patients that did not receive these antibiotics. While the observed lack of changes in the overall microbiota community structure and alpha diversity could be due to exposure of the control group to antibiotics other than 3GC and TZP or to antibiotic-exposed microbiota in the ICU environment more generally, this is an inherent challenge when conducting a human clinical trial in a critical care setting.

### Antibiotic administration altered microbial composition in all three body sites

**Figure 2:**
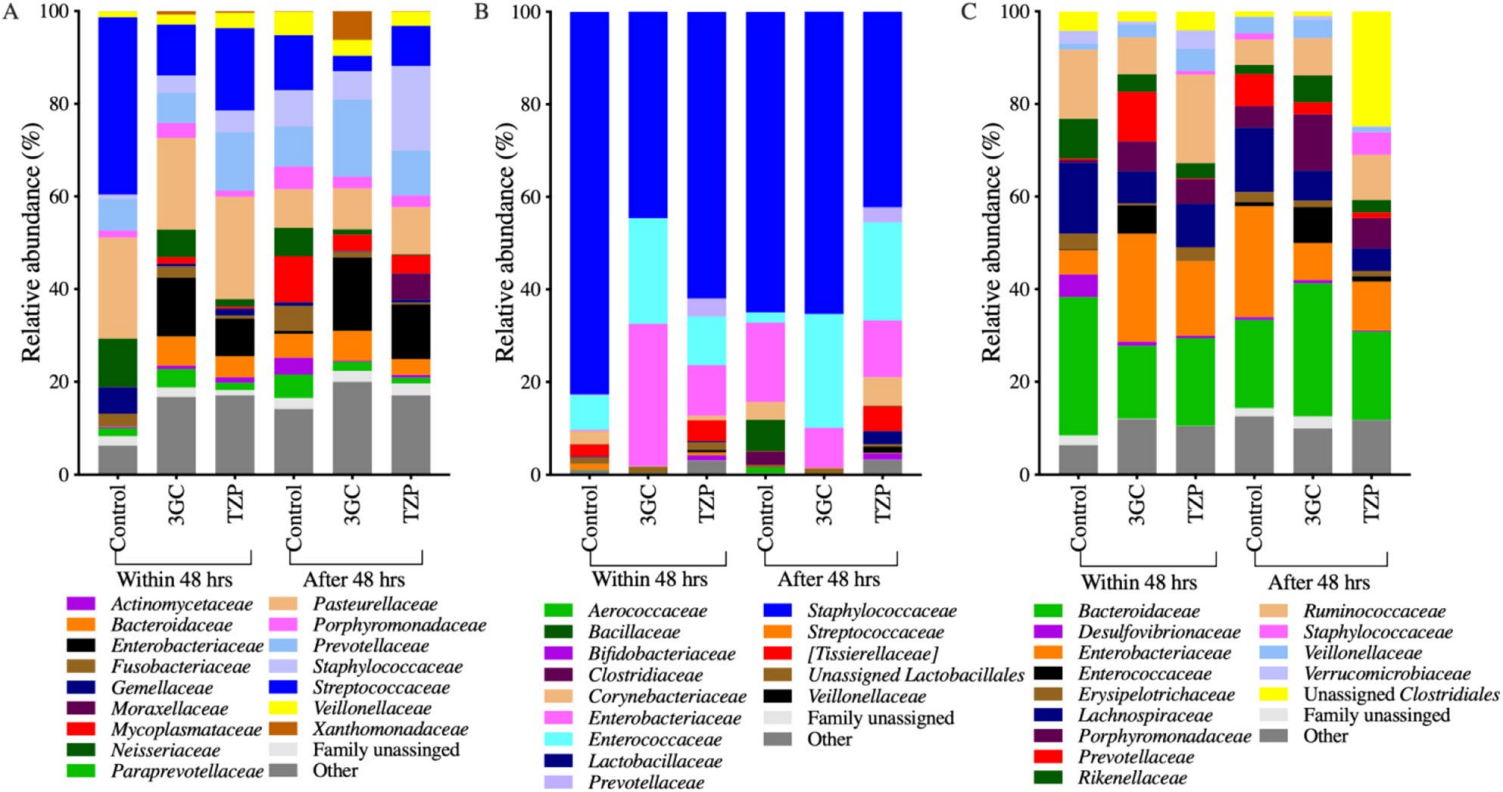
Family level taxonomic composition of the (**A**) endotracheal, (**B**) perineal and (**C**) faecal microbiota samples collected within and after 48 h of antibiotic administration (for 3GC and TZP groups) or admission to the ICU (control group). 3GC-third-generation cephalosporins, TZP-piperacillin/tazobactam, control-no β-lactams. OTUs that were not assigned to a family are categorised as “Family unassigned”. Bacterial families with a relative abundance less than 3% in all three treatments for each body site are grouped as “Other”. Family level taxonomic abundance for each patient is shown in Supplementary Fig. S3 with details on the sample size per treatment group provided in Supplementary Table S1. The relative abundance of the 16S rRNA gene amplicons in the families were determined using QIIME and graphed using GraphPad Prism (version 9, GraphPad Software, USA, www.graphpad.com).

**Figure 3:**
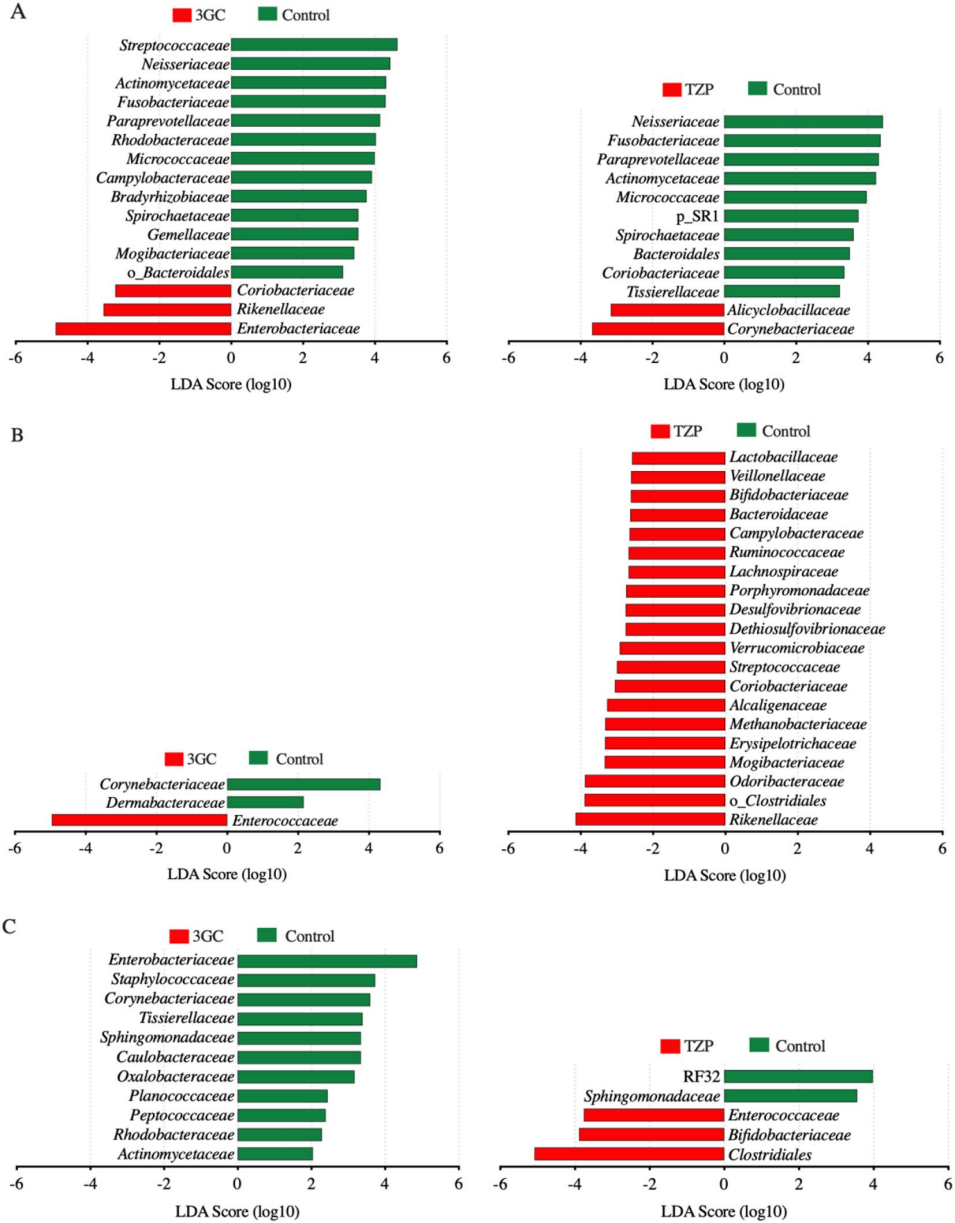
Bacterial families in the (**A**) endotracheal, (**B**) perineal, and (**C**) faecal microbiota showing significantly different abundances in the 3GC and TZP groups compared to the control group after 48 h (3GC-third-generation cephalosporins, TZP-piperacillin/tazobactam, control-no β-lactams). Data were obtained using LEfSe analyses between each antibiotic treatment and the control group. The histograms show the linear discriminant analysis (LDA) scores computed for each bacterial family.

While neither antibiotic treatment had a significant impact on overall microbiota community structure and diversity, there were significant changes in the relative abundance of specific bacteria (Fig. 2). Bacterial families (Fig. 3 and Supplementary Fig. S3) and OTUs (Supplementary Table S3) with significantly different abundances between treatments were identified using linear discriminant analysis effect size (LEfSe) tests comparing each antibiotic treatment with the control group (> 48 h) for each body site. The endotracheal microbiota of patients in the 3GC group had a higher relative abundance of *Enterobacteriaceae* compared to the control group, while administration of TZP resulted in a higher abundance of some typical airway-dwellers, such as *Alicyclobacillaceae* and *Corynebacteriaceae* (Fig. 3A and Supplementary Fig. S3). *Neisseriaceae* were significantly reduced after both TZP and 3GC exposure. *Streptococcaceae* were significantly lower after treatment with 3GC only, while *Fusobacteriaceae* were significantly reduced only after TZP treatment. These are all well-known early colonisers of endotracheal tubes in intubated patients^43^, but a higher abundance of *Enterobacteriaceae* has been associated with respiratory tract infections^38,39^ and increased antibiotic resistance upon administration of 3GC has also been observed in members of this family^44,45^.

Perineal microbiota samples from patients receiving 3GC or TZP treatment had differing abundances of a number of bacterial families compared to the control group (Fig. 3B and Supplementary Fig. S3). *Enterococcaceae* were significantly more abundant after 3GC treatment, but not significantly higher in the TZP treated group in keeping with the weaker antimicrobial activity of 3GC against *Enterococcaceae* than TZP. 3GC exposure was also associated with significantly less commensal bacterial families, *Corynebacteriaceae* and *Dermabacteraceae,* in the perineal microbiota compared to the control group, while the TZP group showed a significantly higher relative abundance of several commensal bacterial families with no significant reduction in the abundance of any major bacterial families. The observed increase in relative abundance of some commensal bacteria following TZP treatment might relate to changes in relative abundance or metabolic activities of TZP-susceptible microbes but further studies are needed to confirm this. The faecal microbiota also seemed to respond differently to the different antibiotics (Fig. 3C and Supplementary Fig. S3). In the 3GC group, several bacterial families showed a significant reduction in abundance compared to the control group with no significant increase in the abundance of any bacterial family. The abundance of *Bifidobacteriaceae, Enterococcaceae* and another unidentified family within the order *Clostridiales* was higher with TZP treatment compared to the control group.

Both antibiotic regimens were associated with a higher abundance of bacterial families that are generally linked with disease (*Enterobacteriaceae* and *Enterococcaceae* in the endotracheal and perineal samples, respectively) but this was statistically significant only for 3GC in this study (Fig. 3 and Supplementary Fig. S3). In the faecal microbiota, this was not apparent where both treatments resulted in a higher abundance of bacterial families generally considered as commensal aerobes or facultative aerobes. Furthermore, compared to the 3GC group, the relative abundance of several bacterial families were different following TZP exposure (Supplementary Fig. S4) and these changes were specific to each body site. For example, the faecal microbiota of patients in the 3GC group had a lower abundance of *Staphylococcaceae* compared to the TZP group. These body site-specific changes in the microbiota were observed across many patients, although substantial individual variation was evident with some samples dominated by one or two taxa; 83% of the perineal samples and 13% each of the endotracheal and faecal samples were dominated by one bacterial family which contributed > 70% to total microbial abundance (Supplementary Fig. S3). Observed heterogeneity in the response of the microbiota to antibiotics could relate to differences in underlying pathologies, age, gender, stress associated with critical illnesses and other lifestyle differences. Future studies with a larger sample size will be essential in investigating the effect of these factors on how the microbiota responds to antibiotic exposure in critical care.

LEfSe analyses conducted at the OTU level showed significant variations after both antibiotic exposures (Table 1 and Supplementary Table S3). While most of these changes showed similar trends as observed at the family level, some specific changes were only observed at the OTU level. For example, the relative abundance of OTUs in families *Lachnospiraceae* and *Ruminococcaceae* appeared to be more strongly represented in the TZP-exposed perineal microbiota compared to the control group, while the 3GC-exposed faecal microbiota had a higher abundance of six OTUs within *Ruminococcaceae* (mostly *Faecalibacterium prausnitzii*) and in *Bacteroides* compared to the control group*,* with relative reductions in the abundance of OTUs within *Enterococcus* and *Staphylococcus* genera. These results demonstrate a clear and distinct impact of short-term administration of 3GC and TZP on the endotracheal, perineal and faecal microbiota of critically ill patients. Sampling microbiota following critical care will be a useful extension to the current study in elucidating associations between microbiota recovery following intensive care and antibiotic exposure.

**Table 1:** The number of OTUs with significantly different abundances in each antibiotic treatment compared to the control group.

Body site	3GC		TZP	
	Higher	Lower	Higher	Lower
Endotracheal	12	73	3	54
Perineal	6	2	54	3
Faecal	17	48	21	34

Data obtained through LEfSe analyses between the relative abundance of bacterial OTUs in each antibiotic treatment (3GC-third-generation cephalosporins, TZP-piperacillin/tazobactam) and control group (no β-lactams) for each body site.

### Culture-based analysis of bacterial antibiotic resistance showed no significant change upon antibiotic exposure

To identify the presence of specific culturable opportunistic pathogens (predominant clones) and changes in their antibiotic resistance phenotypes, we performed culture-based microbiological analysis (on selective media) on samples from a subset of patients for whom faeces were collected both at ICU admission (< 48 h) and after antibiotic administration (> 48 h). As expected for such powerful broad-spectrum antibiotics, particularly in endotracheal and perineal sites, 40% of samples failed to yield any detectable bacterial growth after overnight aerobic incubation on specific isolation media (Supplementary Table S4). Previous studies have indicated that 3GC can increase the risk of colonisation by extended-spectrum β-lactam resistant strains^3^, but the culturable microbes in this study were not significantly enriched for major aerobic pathogens (Supplementary Table S5). For specimens with aerobic bacterial growth on day 1, pathogenic species were detected with approximately the same frequency in all treatment groups and, in accordance with previous results^3,33^, there was no immediate evidence of increased resistance after either antibiotic treatment (Supplementary Tables S5 and S6). Statistical analysis of the collected data did not find significant associations in the frequency of methicillin-resistant *Staphylococcus aureus, Pseudomonas aeruginosa*, or aerobic Gram-negative bacteria between treatments and within treatments, likely due to the small sample size.

Our results demonstrate no significant change in the presence of specific pathogens and their antibiotic resistance after short-term exposure (< 11 days) to 3GC or TZP, but this examination was limited to specific culturable bacteria and the number of samples per treatment group was low. While the average length of stay is typically less than a week in ICUs of this type, the impact of longer-term exposure to these antibiotics would be a useful future extension to this study. Future larger scale studies with quantitative approaches to detect pathogens and experimental designs to analyse the gut resistome are important in obtaining a comprehensive overview of the impact of 3GC and TZP on the relative abundance of opportunistic pathogens and levels of antibiotic resistance, respectively, and are currently in progress.

## Conclusions

Administration of either of two broad-spectrum antibiotics, 3GC or TZP, resulted in significant changes in the endotracheal, perineal and faecal microbiota of ICU patients. The relative abundance of the *Enterobacteriaceae* and *Enterococcaceae*, which include some of the main nosocomial antimicrobial-resistant pathogens^46^, was significantly higher in the endotracheal and perineal microbiota only after 3GC exposure, while TZP treatment was associated with an increase in the abundance of bacteria generally considered as commensals, particularly in the perineal microbiota. TZP treatment was associated with an increase in relative abundance of *Enterococcaceae* in the faecal microbiota, demonstrating a clear body site-specific impact of the two antibiotic regimens.

Third-generation cephalosporins are widely believed to be more injurious to the microbiota than many other antibiotics with comparable safety and efficacy profiles and clinical indications in critically ill patients. Consequently, restriction of their use is a major goal of antibiotic stewardship programs and is associated with reduced costs and reduced rates of infection due to resistant pathogens, with the benefits being most marked in intensive care settings^47^. Despite the small sample size and the complexity of these responses in inherently dysbiotic environments, our ‘real world’ data support such clinical observations, provide direct information on how these different antibiotic treatments affect the microbiota and underline the value of larger scale studies.

## Methods

### Patient recruitment

The target population for this study included critically ill, mechanically ventilated patients who had not received the specific antibiotics (3GC or TZP) for at least two weeks prior to ICU admission. The three study cohorts were: (1) patients receiving a third-generation cephalosporin (3GC; ceftriaxone or cefotaxime); (2) patients receiving piperacillin/tazobactam (TZP); Tazocin); and (3) patients who were critically ill and mechanically ventilated but did not receive either TZP or 3GC during ICU stay (control group). The latter group was recruited from patients presenting with trauma, acute cardiac or cerebral ischemic events, severe asthma or status epilepticus, and who had not received antibiotics in the previous two weeks.

### Specimen collection and storage

Specimens were collected over a two-year time span (2015–2017) at two hospitals in western Sydney (Westmead and Nepean ICUs), Australia. Perineal swabs and endotracheal aspirates were collected from patients as a part of routine clinical praxis on day 1 (24 h from the start of antibiotic treatment), day 3 (72 h from the start of antibiotic treatments), day 5 and day 7 (if the patient was still in the ICU). Faecal specimens were collected each time a patient passed a bowel motion. Antibiotic effects were determined by comparison of samples prior to (< 48 h) and after (> 48 h) antibiotic treatment. Faecal samples were immediately frozen at − 20 °C, and endotracheal aspirates and perineal swabs were refrigerated (2–8 °C). After collection at the approved clinical sites, the specimens were transferred to the laboratories for further analysis.

### Microbiota analysis

Total microbial DNA was extracted from endotracheal aspirates, perineal swabs and faeces using a MoBio PowerSoil DNA extraction kit (MoBio, Australia) according to the manufacturer’s instructions. The V4 variable region of the 16S rRNA genes in extracted DNA was amplified using the 515F-806R primer pair with custom barcodes for Illumina MiSeq sequencing^48,49^. Sequencing of the amplicons was performed on an Illumina MiSeq v2 platform (2 × 250 bp). Library preparation and sequencing were performed at the Ramaciotti Centre for Genomics (UNSW, Sydney, Australia).

Demultiplexed raw sequence data were processed using Quantitative Insights Into Microbial Ecology (QIIME) software (version 1.9.1)^50^. Full length and high quality (− q 19 and with other default parameters) reads were used to determine operational taxonomic units (OTUs) at 97% similarity using an open-reference protocol against the Greengenes database (version 13_8)^51^. A total of 19,638,026 reads (mean 93,291 ± 56,089) were obtained for a total of 231 samples prior to filtering out OTUs with less than 0.005% reads. Reads per sample were rarefied to 10,387 reads, nine random samples failed to meet this requirement and were eliminated from further analysis. A total of 222 samples were retained and used for further statistical analysis.

All statistical analyses on the microbiota data were performed using rarefied and filtered OTUs.

### Statistical analysis of microbiota sequence data

The OTU abundances (Log (x + 1) transformed) were used for analysis using PRIMER-7 software package^52^ (default parameters were used unless otherwise stated). Overall microbiota community structure was visualised using multi-dimensional scaling (MDS) plots based on Bray–Curtis similarity metrics of the abundance of the OTUs. Permutational Multivariate Analysis of Variance (PERMANOVA) tests with 9999 permutations were conducted to investigate statistical significance of differences in the microbial community structure between treatments. The Shannon diversity and Simpson’s evenness indices per sample were also determined using PRIMER-7. Statistical significance of the differences in the alpha diversity indices was determined through Tukey’s multiple comparison tests with Geisser-Greenhouse correction using GraphPad Prism (version 9, GraphPad Software, USA, www.graphpad.com).

Bacterial families and OTUs with significantly different abundances between the control group and each antibiotic treatment were identified using Linear discriminant analysis Effect Size (LEfSe, online Galaxy version 1.0) analysis^53^. Separate LEfSe analyses were conducted on samples collected within and after 48 h of antibiotic exposure to determine the differences in the initial microbiota and the impact of antibiotic exposure, respectively. All LEfSe analyses were conducted using antibiotic treatment groups as the subject (no subclasses) and all other default parameters, including Kruskal–Wallis test among classes (*P* < 0.05), Wilcoxon test between classes (*P* < 0.01) and the threshold on the logarithmetic LDA score for discriminative features > 2.0. Detailed information on the number of patients included in each analysis are provided in Supplementary Table S1. False discovery rate analyses on LEfSe outputs (*P* values) were conducted through Benjamini and Hochberg method using GraphPad Prism (version 9, GraphPad Software, USA, www.graphpad.com). Significant differences in the abundance of bacterial families and OTUs that passed an adjusted *P* value of 0.05 are presented.

### Pathogen and *Enterobacteriaceae* identification

Aerobic microbiota was cultured from collected specimens by direct inoculation of 5 mL nutrient broth (NB; Oxoid, UK) and overnight incubation at 37 °C with shaking. Cultures were pelleted by centrifugation (7000×*g*; 10 min), supernatants discarded, and pellets washed in saline and then re-suspended in 1 mL 20% glycerol for long term storage at − 80 °C. Methicillin-resistant *Staphylococcus aureus* and *Pseudomonas aeruginosa*, pathogenic species known to be highly abundant in ICU patients under antibiotic treatment^3^ and predominant Gram-negative enteric bacteria were sub-cultured on species-specific identification media: Brilliance MRSA Agar (Oxoid, UK), Difco *Pseudomonas* Isolation Agar (Becton Dickinson, Sparks, USA), and ChromAGAR supplemented with vancomycin (20 µg/mL, to exclude Gram positive species), respectively. Isolated enteric colonies identified as potential *Escherichia coli* (pink colonies) or *Klebsiella pneumoniae* (blue colonies) were also tested for resistance to extended-spectrum β-lactams by plating on selective media (Brilliance ESBL Agar (Oxoid, UK) and LB agar (Sigma, Australia) supplemented with 2 µg/mL cefotaxime). Bacterial isolates (six colonies per sample) from faecal specimens were also screened on a panel of clinically relevant antibiotics (amikacin; ampicillin; piperacillin/tazobactam; ceftriaxone; ciprofloxacin; gentamicin; meropenem) by automated disc-diffusion assay (BD Kiestra Becton, Dickinson and Co, Drachten, Netherlands). Statistical analysis of significance (*P* ≤ 0.05) was performed using McNemar’s test and regression models fitted on the data.

### Ethics declaration

All experimental procedures and protocols were reviewed and approved by the Human Research Ethics Committees of Nepean (LNR/14/NEPEAN/72) and Westmead (LNRSSA/14/WMEAD/313) hospitals. All methods were performed in accordance with the relevant guidelines and regulations. The study was conducted under a waiver of consent, with the auspices of the above-mentioned Human Research Ethics Committees.

## Supplementary Information

Supplementary Information 1.

Supplementary Table S1.

Supplementary Table S2.

Supplementary Table S3.

## Data Availability

The 16S rRNA gene sequence data generated and analysed in this study are available on the Sequence Read Archive under accession number PRJNA683977.
