# Supplementary material for: Third generation cephalosporins and piperacillin/tazobactam have distinct impacts on the microbiota of critically ill patients
Source: Sci Rep. 2021 Mar 31;11:7252. doi: 10.1038/s41598-021-85946-4 (PMC8012612; doi:10.1038/s41598-021-85946-4)
Supplement: Supplementary file 1 — Supplementary Information 1. [file 41598_2021_85946_MOESM1_ESM.pdf]

Third generation cephalosporins and piperacillin/tazobactam have distinct impacts on the microbiota of critically ill patients

Hasinika K.A.H. Gamage[1,†], Carola Venturini[2,†], Sasha G. Tetu[1], Masrura Kabir[2,7], Vineet Nayyar[3], Andrew N. Ginn[2,4], Belinda Roychoudhry[2], Lee Thomas[2], Mitchell Brown[4], Andrew Holmes[5], Sally R. Partridge[2], Ian Seppelt[6], Ian T. Paulsen[1,*], Jonathan R. Iredell[2,*]

Supplementary Figures and Tables

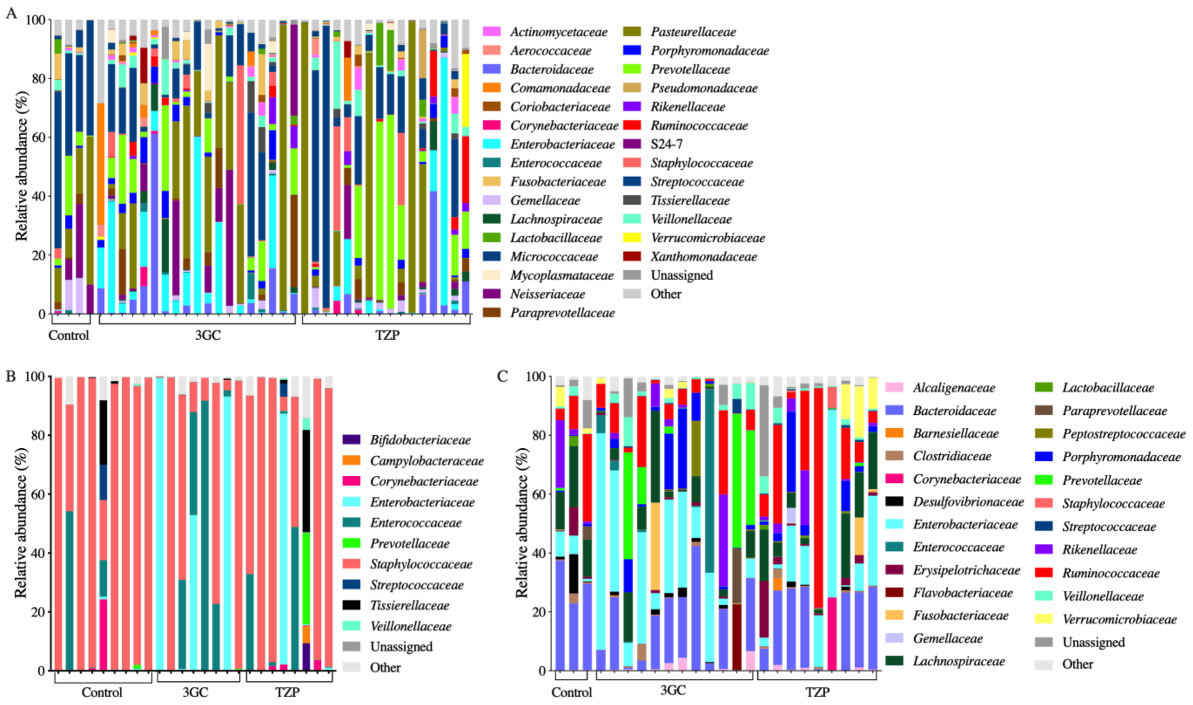

Figure S1 Family level taxonomic composition of the **(A)** endotracheal, **(B)** perineal, and **(C)** faecal microbiota of each patient. The microbial abundance of samples collected within 48 hours of 3GC or TZP administration or admission to the ICU was used as the baseline microbiota composition of each patient, (3GC- third-generation cephalosporins, TZP- piperacillin/tazobactam, control- no β-lactams). The relative abundance of the 16S rRNA gene amplicons in the families were determined using QIIME and graphed using GraphPad Prism (version 9, GraphPad Software, USA, www.graphpad.com). OTUs that were not assigned to a family are categorised as "Unassigned". Bacterial families with a relative abundance less than 3% in all three treatments for each body site are grouped as "Other".

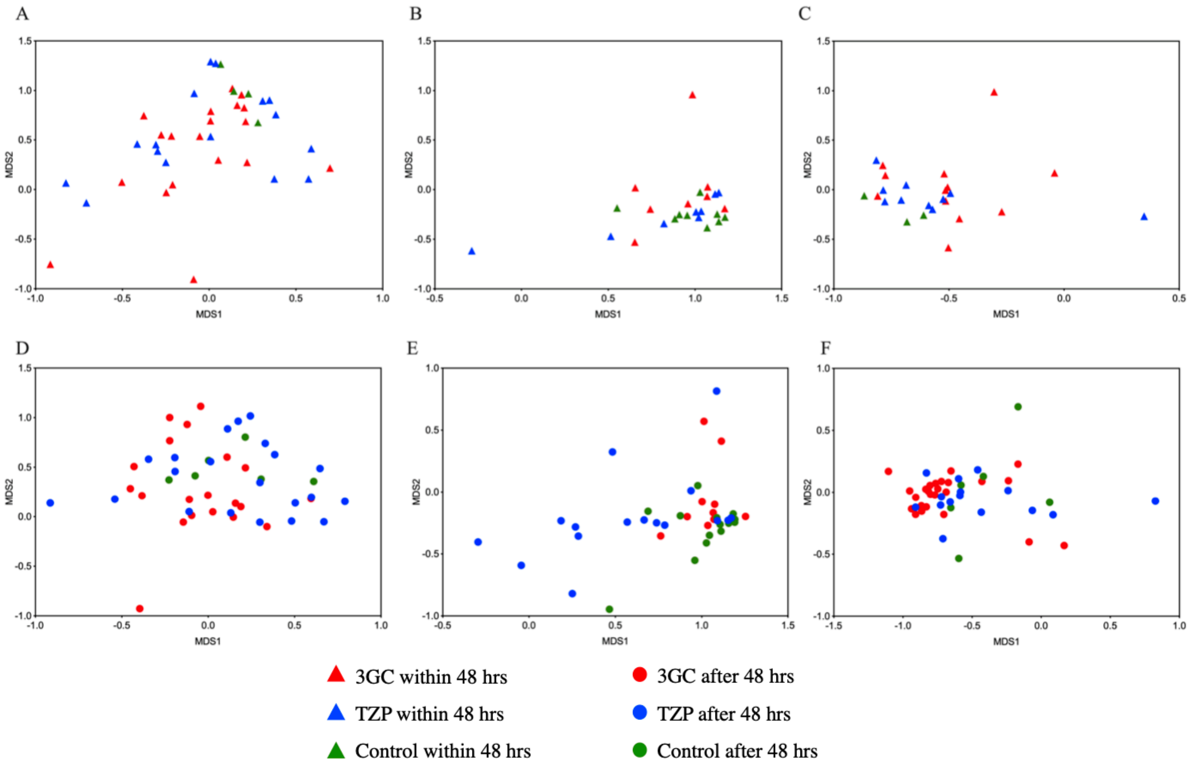

Figure S2 Ordination of microbiota samples collected within and after 48 hours of antibiotic administration (for 3GC and TZP groups) or ICU admission (for control group). Data are presented as multidimensional scaling (MDS) plots based on Bray Curtis similarity metrics of OTU abundance. Samples collected from the **(A)** endotracheal microbiota (<48 hrs), **(B)** perineal microbiota (<48 hrs), and **(C)** faecal microbiota (<48 hrs) and those collected from the **(D)** endotracheal microbiota (>48 hrs), **(E)** perineal microbiota (>48 hrs), and **(F)** faecal microbiota (>48 hrs) are shown. The overall microbiota community structure in the 3GC (third-generation cephalosporins) and TZP (piperacillin/tazobactam) groups were compared against the control group (no β-lactams).

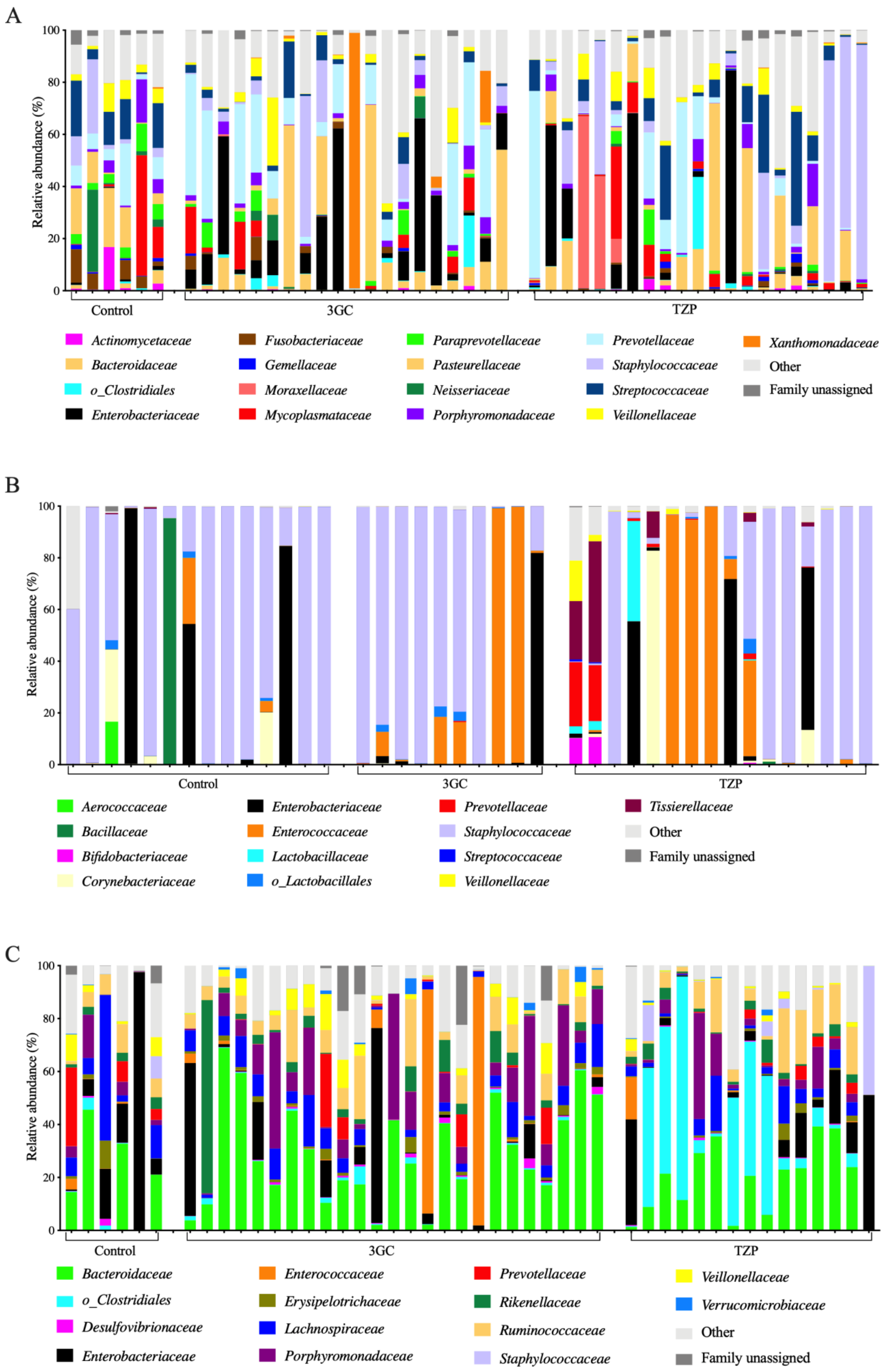

Figure S3 Family level taxonomic composition of the **(A)** endotracheal, **(B)** perineal and **(C)** faecal microbiota samples collected after 48 hours of 3GC or TZP administration or admission to the ICU, (3GC- third-generation cephalosporins, TZP- piperacillin/tazobactam, control- no β-lactams). Data is shown for each patient categorised based on treatment group. OTUs that were not assigned to a family are categorised as "Family unassigned". Bacterial families with a relative abundance less than 3 % in all three treatments for each body site are grouped as "Other". The relative abundance of the 16S rRNA gene amplicons in the families were

determined using QIIME and graphed using GraphPad Prism (version 9, GraphPad Software, USA, www.graphpad.com).

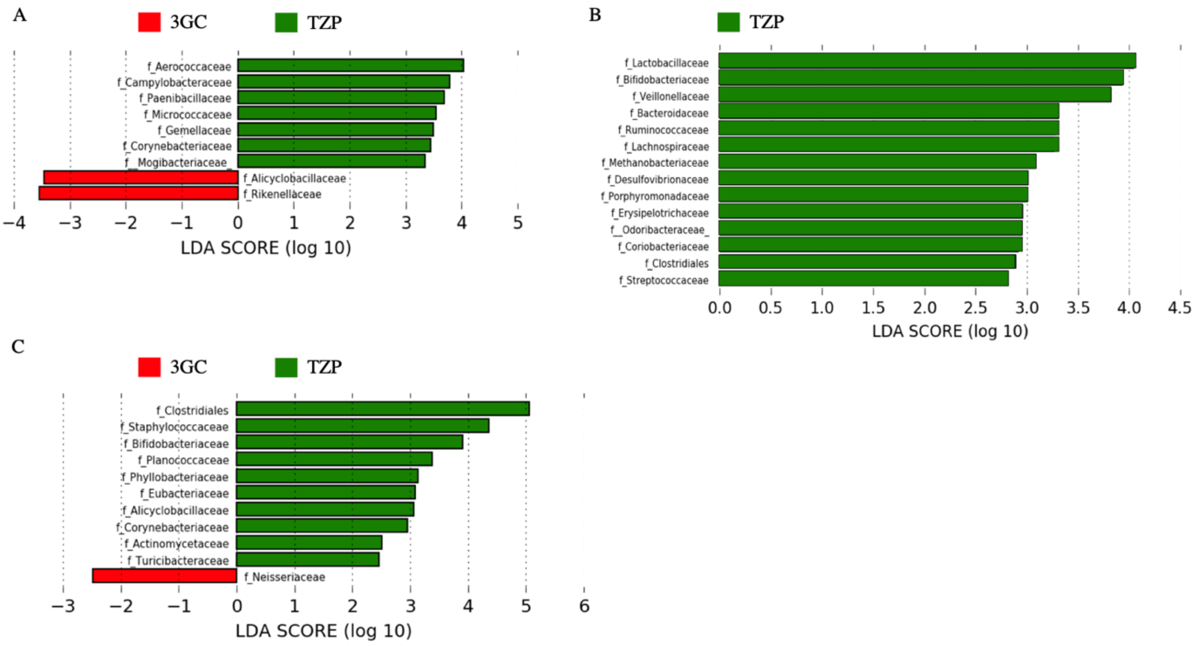

Figure S4 Bacterial families in the **(A)** endotracheal, **(B)** perineal, and **(C)** faecal microbiota showing significantly different abundances between the 3GC and TZP groups after 48 hours of antibiotic exposure (3GC- third-generation cephalosporins and TZP- piperacillin/ tazobactam). The histograms show the linear discriminant analysis (LDA) scores computed for each bacterial family. LEfSe analyses were performed with the following parameters, Kruskal-Wallis test among classes (*P* < 0.05), Wilcoxon test between classes (*P* < 0.01) and the threshold on the logarithmetic LDA score for discriminative features > 2.0.

Table S1 Number of samples collected per treatment group on each day.*
(Provided as an Excel file)
***(A)** A summary of the number of samples collected for each treatment group within and after 48 hours of ICU admission. Details on patient identifiers and the number of **(B)** endotracheal, **(C)** perineal and **(D)** faecal microbiota samples collected on each day are provided.

Table S2 Bacterial families that were found to be significantly differentially abundant in the 3GC and TZP groups compared to the control group within the first 48 hours of antibiotic administration.*
(Provided as an Excel file)
*Data was obtained based on LEfSe analyses between 3GC vs control group and TZP vs control group in the **(A)** endotracheal **(B)** perineal and **(C)** faecal microbiota. LEfSe analyses were performed with the following parameters: Kruskal-Wallis test among classes ($P < 0.05$), Wilcoxon test between classes ($P < 0.01$) and the threshold on the logarithmetic LDA score for discriminative features > 2.0. 3GC- third-generation cephalosporins, TZP- piperacillin/tazobactam, control- no β-lactams.

Table S3 The relative abundance of bacterial OTUs that were found to be significantly differentially abundant between the three treatments after 48 hours of admission.*
(Provided as an Excel file)
*Data was obtained based on LEfSe analyses between **(A)** 3GC vs control group in the endotracheal microbiota, **(B)** TZP vs control group in the endotracheal microbiota, **(C)** 3GC vs control group in the perineal microbiota, **(D)** TZP vs control in the perineal microbiota, **(E)** 3GC vs control in the faecal microbiota and **(F)** TZP vs control in the faecal microbiota. LEfSe analyses were performed with the following parameters: Kruskal-Wallis test among classes ($P < 0.05$), Wilcoxon test between classes ($P < 0.01$) and the threshold on the logarithmetic LDA score for discriminative features > 2.0. 3GC- third-generation cephalosporins, TZP- piperacillin/tazobactam, control- no β-lactams.

Table S4 Specimens used for routine microbiology testing (number of tested samples with overnight growth/total number tested is shown).*

**Specimen**	**Endotracheal**		**Perineal**		**Faecal**		**All**
	<48 hrs	>48 hrs	<48 hrs	>48 hrs	<48 hrs	>48 hrs	
**TZP**	4/11<br>36%	3/11<br>27%	3/10<br>30%	7/9<br>78%	3/5<br>60%	2/8<br>25%	22/54<br>40%
**3GC**	3/13<br>23%	2/13<br>15%	3/5<br>60%	2/5<br>40%	7/10<br>70%	9/12<br>75%	26/58<br>45%
**Control**	2/11<br>18%	2/6<br>33%	2/7<br>28.5%	5/6<br>83%	(1/3)^<br>33%^	(2/2)^<br>100%^	14/35<br>40%
total	9/35<br>26%	7/30<br>23%	8/22<br>36%	14/20<br>70%	7/10<br>70%	9/12<br>75%	**54/129**<br>**42%**

*3GC- third-generation cephalosporins, TZP- piperacillin/tazobactam, control- no β-lactams; ^only rectal swabs obtained from control cohort

Table S5 Identification of specific opportunistic pathogens in human specimens using microbiological culturing.*

		Control			TZP			3GC		
		**GNB**	**MRSA**	**Pa**	**GNB**	**MRSA**	**Pa**	**GNB**	**MRSA**	**Pa**
**Endotracheal**	<48hr	2/4	0/4	0/4	5/12	1/12	0/12	6/15	7/15	0/15
	>48hr	1/5	2/5	0/5	2/16	0/16	2/16	3/18	6/18	0/18
**Faecal**	<48hr	0/1	1/1	0/1	3/5	0/5	0/5	8/13	1/13	0/13
	>48hr	0/2	2/2	0/2	2/8	1/8	0/8	13/22	3/22	1/22
**Perineal**	<48hr	2/7	1/7	0/7	3/11	2/11	1/11	3/5	1/5	0/5
	>48hr	8/15	0/15	0/15	12/18	1/18	1/18	3/7	3/7	1/7

*GNB indicates Gram negative bacteria (*E. coli* and *K. pneumoniae*) identified on chromogenic agar (CHROMAgar) as pink or blue colonies; MRSA, multidrug resistant *Staphylococcus aureus*; Pa, *Pseudomonas aeruginosa*. Values shown as number of positive samples versus total number of samples processed. Only patients with specimens collected both on day 1 (admission to ICU) and on at least one subsequent day were included in this analysis.

Table S6 Antibiotic resistance phenotypes for bacterial isolates recovered from faeces.

Isolate	Day	Treatment	Organism	Antibiotic Resistance*
N05F1	1	control	*E. coli*	Ap
N05F3	3	control	*E. coli*	Ap Gm CTX
N05F3	3	control	*E. coli*	Ap Gm TZP(i) CTX
N14F1	1	TZP	*E. coli*	Ap Gm
N14F3	3	TZP	*E. coli*	Ap Gm CTX
N22F2	1	TZP	*E. coli*	Ap
N22F11	11	TZP	*E. coli*	Ap
N01F1	1	CRO	*E. coli*	Ap CTX
N07F1	1	CRO	*E. coli*	Cip
N07F1	1	CRO	*K. oxytoca*	Ap
N09F1	1	CRO	*E. coli*	Cip
N10F3	3	CRO	*E. coli*	Cip
N13F2	1	CRO	*E. coli*	none
N13F4	3	CRO	*E. coli*	none
N21F1	1	CRO	*E. coli*	Ap
N21F3	3	CRO	*E. coli*	Ap
N21F1	1	CRO	*K. pneumoniae*	Ap
N21F3	3	CRO	*K. pneumoniae*	Ap
N21F3	3	CRO	*K. pneumoniae*	Ap
N24F1	1	CRO	*E. coli*	none
N24F3	3	CRO	*E. coli*	none
N24F3	3	CRO	*E. coli*	none
W35F2	1	CRO	*E. coli*	Cip

*(i), intermediate.
